# An exopolysaccharide from *Bacillus subtilis* alleviates airway inflammatory responses via the NF-κB and STAT6 pathways in asthmatic mice

**DOI:** 10.1042/BSR20212461

**Published:** 2022-01-28

**Authors:** Lingxiu Zhang, Huilan Yi

**Affiliations:** 1 School of Life Science, Shanxi University, Taiyuan 030006, China; 2 College of Environment and Resource Sciences, Shanxi University, Taiyuan 030006, China; 3 Department of Biology, Xinzhou Teachers University, Xinzhou 034000, China

**Keywords:** airway inflammation, asthma, Bacillus subtilis, exopolysaccharide, NF-κB pathway, STAT6 pathway

## Abstract

*Bacillus subtilis* is an intestinal probiotic for immune homeostasis and its exopolysaccharide (EPS) is known to possess anti-inflammatory and antioxidant properties. The underlying mechanisms are not yet fully understood. In the present study, we investigated the effects of the EPS (50, 100, 200 mg/kg) on airway inflammation in asthmatic mice. Our results showed that EPS treatment of asthmatic mice significantly alleviated pathological damage in the lungs, remarkably decreased the counts of total inflammatory cells including lymphocytes, and eosinophils in the bronchoalveolar lavage fluid (BALF) and reduced indexes of oxidative damage. Moreover, the expression of type II T-helper cell (Th2) cytokines (interleukin- (IL)4 and -5) subsequent to EPS treatment was found to be dramatically down-regulated in a concentration-dependent manner. Additionally, the EPS treatment reduced JAK1, STAT6 and nuclear factor-κB (NF-κB) expression in the lungs of asthmatic mice. Taken together, these results suggest that the EPS from *B. subtilis* alleviates asthmatic airway inflammation, which involves the reduction in reactive oxygen species (ROS) and the down-regulation of the STAT6 and NF-κB inflammatory pathways, which can further reduce Th2 cytokine expression and eosinophilic inflammation. Thus, our findings provide a potential mechanism through which the EPS mitigates asthma, suggesting that the EPS could be a potential source of an anti-asthmatic drug.

## Introduction

Currently, asthma affects more than 339 million people and has become a serious socioeconomic burden and a major global issue [1,2]. It is an allergy-induced (e.g., by house dust, pollen, animal dander) and a partly type II T-helper cell (Th2)-mediated inflammatory disease characterized by pulmonary eosinophilia, mucus hypersecretion, and airway hyperresponsiveness (AHR) [3,4]. Airway epithelial cells, dendritic cells, T regulatory cells, T helper (Th) cells, granulocytes, and activated macrophages mediate allergic asthma [5]. A Th1/Th2 imbalance plays an important role in the pathogenesis of asthma and the Th2-associated cytokines interleukin (IL)-4 and -5 are released abnormally to trigger an inflammatory response [6]. Furthermore, the nuclear factor-κB (NF-κB) and STAT6 pathways are also involved in inflammatory and immune regulation of the innate and adaptive immune system [7–9].

Although drugs used for asthma, such as inhaled steroids and bronchodilators, can control symptoms well, certain side effects have been demonstrated; e.g., tachycardia, osteoporosis and influence on the development of children and adolescents [10]. *Bacillus subtilis*, a probiotic, provides benefits to hosts, such as the secretion of antimicrobials and the prevention of inflammatory diseases by regulation of the innate and T cell-mediated immune responses [11]. The exopolysaccharide (EPS) from *B. subtilis* has become a very popular research topic due to its antioxidant activity, anti-inflammatory efficacy, and antitumor activity [12–14]. *B. subtilis* can prevent diarrheal disease and acute colitis; educate the immune system; and elicit allergic eosinophilia by restraining the function of dendritic cells [15,16]. The oligosaccharide produced by *B. subtilis* suppresses asthma through modulation of Th1/Th2-related cytokines [17]. EPS from *B. subtilis* can elicit allergic eosinophilia, inhibiting dendritic cell function [18]. The anti-inflammatory mechanism of EPS from *B. subtilis* may be associated with M2 macrophages or T lymphocytes [19]. However, the detailed mechanisms of the EPS *vis-à-vis* regulation of immune system and its anti-inflammatory properties have not been fully established.

In the present study, the effect of the EPS from *B. subtilis* on ovalbumin (OVA)-induced asthma in mice was examined. We investigated pulmonary histopathological changes, the proportion of inflammatory cell numbers in the bronchoalveolar lavage fluid (BALF), indexes of oxidative damage and mRNA levels of mucin 5AC (MUC5AC), IL-4 and IL-5. We then investigated the components involved in the STAT6 and NF-κB pathways and the IgE level. These findings suggested a potential mechanism through which the EPS may attenuate asthma in mice and indicated that the EPS from *B. subtilis* was capable of alleviating inflammatory responses in asthma.

## Materials and methods

### Preparation of the EPS from *B. subtilis*

*B. subtilis* xztubd1 (GenBank: MG458322.1) was used for production of the EPS. The activating bacteria were cultured in a 10-l automatic fermenter at 300 rpm and 37°C for 3 days. The fermentation broth was centrifuged at 5000×***g*** for 15 min; the supernatant was concentrated in a rotary evaporator, then mixed with 4 volumes of prechilled ethanol, and allowed to stand for 24 h at 4°C. The precipitation was freeze-dried to a powder, resuspended, then treated with DNase and RNase. The EPS was further purified on a SephadexG-75 column and quantified by dry weight.

### Animal model and treatment protocols

Six-week-old Kunming male mice (18–20 g) were purchased from the experimental animal center of the Tumor Hospital of Shanxi (China). The animals were housed in the animal laboratory of School of Life Science in Shanxi University and had free access to abundant food and sterile water in a sterile environment. All animal experiments were approved by the Institutional Animal Care and Use Committee of Shanxi University (sx20200825k38520) and were conducted in strict accordance with Chinese National Standards (GB/T35823-2018). Mice were anesthetized by sodium pentobarbital and killed by cervical dislocation.

**Figure 1:**
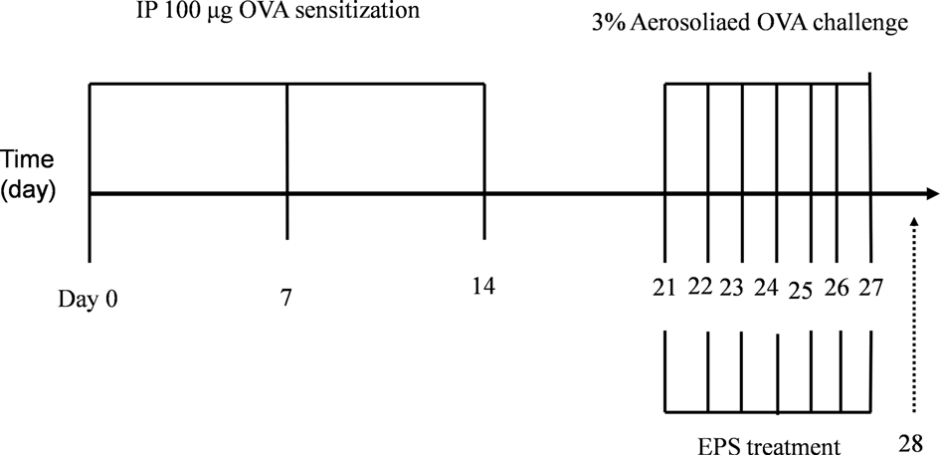
Allergen sensitization, challenge, and the EPS pre-treatment protocols in mice

The mice were assigned at random to the following six groups, each consisting of six mice: control, OVA, OVA+1 mg/kg/day Dexa (dexamethasone), OVA+EPS (50 mg/kg/day), OVA+EPS (100 mg/kg/day), and OVA+EPS (200 mg/kg/day). Mice in the OVA treatment group were sensitized with 200 μl saline (containing 100 μg OVA and 2 mg aluminum hydroxide) by intraperitoneal injections on days 1, 7 and 14, then challenged with aerosolized 3% OVA for 30 min daily from days 21 to 27. During this period, the OVA+EPS groups received dexamethasone and EPS of different concentrations dissolved in sterile water through oral administration 1 h prior to the OVA challenge [4]. The sensitization, challenge, and treatment protocols are summarized in Figure 1.

### Lung histological examination and inflammatory cell assessment

Mice were anesthetized by an intraperitoneal injection of sodium pentobarbital (50 mg/kg) and killed on the 28^th^ day. The right main bronchus was ligated and the left lung was washed three times with sterile saline (a total of 1 ml). The BALF was precipitated and suspended in 0.1 ml saline. All leukocytes were counted using a hemocytometer, and classified by Wright–Giemsa-stained smears. Additionally, the middle lobe of the right lung was fixed in 10% neutral formalin, embedded in paraffin, sliced into 5-μm-thick sections, and observed for histopathological changes using Hematoxylin–Eosin staining [20]. The remaining the right lung lobes were immediately frozen and stored at −80°C for subsequent analysis.

### Assessment of oxidative damage indexes in the lungs

Mouse lungs (0.1 g) were added to a nine-fold volume of cold normal saline and homogenized. The sample was centrifuged at 4000×***g*** for 10 min at 4°C and the supernatant was used to test the oxidative indexes. The hydrogen peroxide (H_2_O_2_), malondialdehyde (MDA) content, and the superoxide dismutase (SOD) and glutathione peroxidase (GSH-PX) activities were measured using assay kits as per the manufacturers’ instructions (Nanjing Jiancheng Bioengineering Institute, China).

### Real-time quantitative polymerase chain reaction

As per the manufacturer's instructions, total RNA was extracted from the lung tissue, then quantified by the measurement of optical density using a spectrophotometer (Eppendorf, Germany). The cDNA was synthesized using the PrimeScript™ RT Reagent Kit (TaKaRa Bio, Japan). The expression of the selected genes was measured using a real-time quantitative polymerase chain reaction (PCR) system (CFX96 Touch; Bio-Rad, Hercules, CA, U.S.A.). The sequences of the primers used are shown in Table 1. The 2^−ΔΔ*C*_t_^ method was adopted to analyze the mRNA levels, and β-actin was used as the internal control for normalization.

**Table 1:** Primer sequences used for quantitative PCR

Gene	Primer sequence (5′–3′)
*JAK1*	ACGCTCCGAACCGAATCATC
	GTGCCAGTTGGTAAAGTAGAACC
*STAT6*	CCTGGTCGGTTCAGATGCTTT
	GTGCGGCAAGATGCTGTTTC
Nuclear factor κB p65	CCACGATCTGTTTCCCCTCAT
	TGATCTCCACATATGGCCCAG
*IL-4*	CCCCAGCTAGTTGTCATCCTG
	CAAGTGATTTTTGTCGCATCCG
*IL-5*	GCAATGAGACGATGAGGCTTC
	GCCCCTGAAAGATTTCTCCAATG
*MUC5AC*	AGGGCTCTGTGACAACTACC
	TGGGGTGTGGGTAGAAGAAC
*β-Actin*	GATTACTGCTCTGGCTCCTA
	ATCGTACTCCTGCTTGCTGA

### Enzyme-linked immunosorbent assay

Levels of total serum IgE were measured using commercially available enzyme-linked immunosorbent assay (ELISA) kits per the manufacturer’s instructions (R&D Systems, U.S.A.). A Multifunctional Microplate Reader (MD SpectraMax M5, U.S.A.) was used to measure the absorbance.

### Statistical analysis

Statistical analysis was conducted using IBM SPSS Statistics 20 (IBM, Armonk, NY, U.S.A.) including significance testing and one-way analysis of variance by Fisher LSD and Dunnett’s multiple comparison tests. All experimental data were expressed as the mean ± standard error. Statistical significance was defined as *P*<0.05.

## Results

### Histopathological changes in the lung

**Figure 2:**
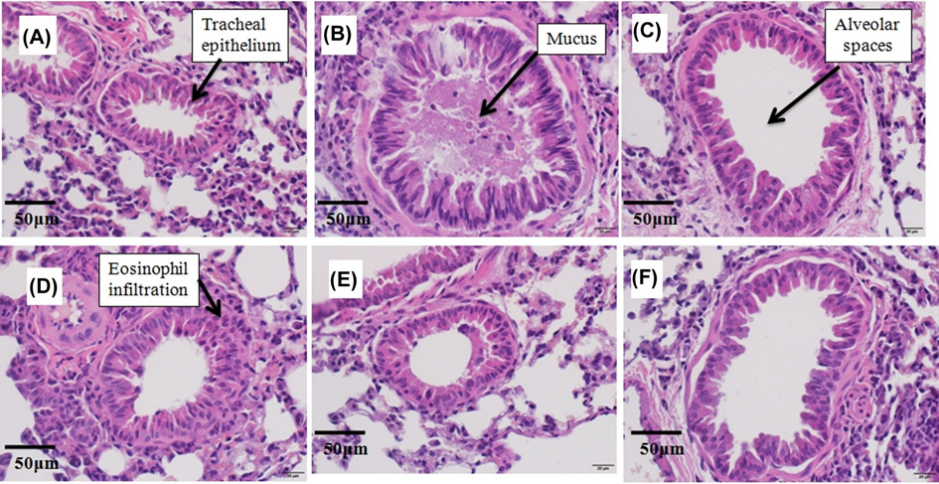
Hematoxylin–Eosin-stained sections of pathological lung tissue (**A**) Control group, (**B**) OVA-induced group, (**C**) Dexa group, (**D**) OVA+50 mg/kg EPS, (**E**) OVA+100 mg/kg EPS, and (**F**) OVA+200 mg/kg EPS treatment. Magnification: 400×. Scale bars = 50 μm.

In order to confirm the effect of the EPS on the alleviation of inflammation in the lungs of asthmatic mice, we examined histopathological changes in the lung tissues in the six treatment groups by HE staining and performed a pathologic analysis to reveal the mechanism. In the control group, no histopathological abnormalities were found (Figure 2A). OVA-sensitized mice showed induced bronchial epithelial thickening, diminished alveolar spaces, clear inflammatory cell infiltration, and marked mucus secretion (Figure 2B). Remarkable relief of airway inflammation was observed in the lungs of mice in the dexamethasone and OVA+EPS treatment, such as significant reduction in eosinophil infiltration, restoration of airway stenosis, and airway mucus secretion (Figure 2C–F). The results showed that the EPS definitely reduced asthmatic airway inflammation at the histological level.

### Inflammatory cell counts in the BALF

**Figure 3:**
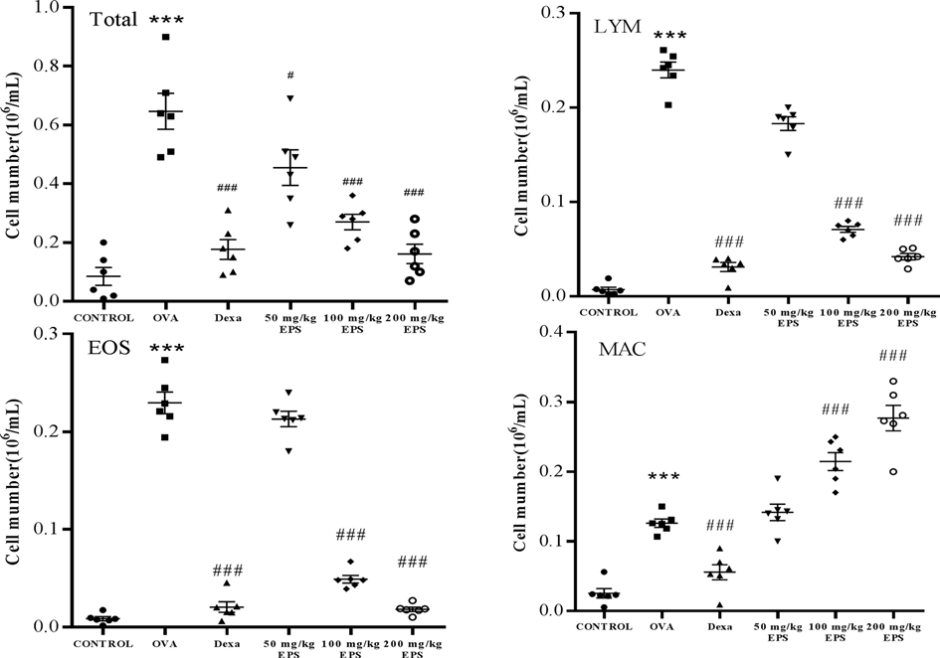
Effects of dexamethasone and the EPS on total number of inflammatory cells and numbers of different types of inflammatory cells in the BALF from mice in each group Total number of inflammatory cells and numbers of different types of inflammatory cells. Data are expressed as the mean ± standard error (*n*=6). ***P<0.001 versus control; ^#^*P*<0.05, ^###^*P*<0.001 versus the OVA group. Abbreviations: EOS, eosinophil; LYM, lymphocyte; MAC, macrophage.

To confirm whether the EPS relieved airway inflammation and which inflammatory cells were regulated in the anti-inflammatory process, we examined the counts of the total inflammatory cells and further determined the number of lymphocytes, eosinophils, and macrophages in the BALF. As shown in Figure 3, the number of inflammatory cells was observably higher in the OVA group than in the control. Treatment with dexamethasone and OVA+EPS markedly diminished the number of inflammatory cells, lymphocytes, and eosinophils in the BALF in a concentration-dependent manner, however, the counts of macrophages in the OVA+EPS treatment significantly increased compared with the OVA group. These results suggest that the alleviation of asthmatic eosinophilic inflammation by EPS treatment may be linked to the prevention of lymphocyte inflammatory responses by anti-inflammatory macrophages.

### Oxidative stress indexes in the lungs

**Figure 4:**
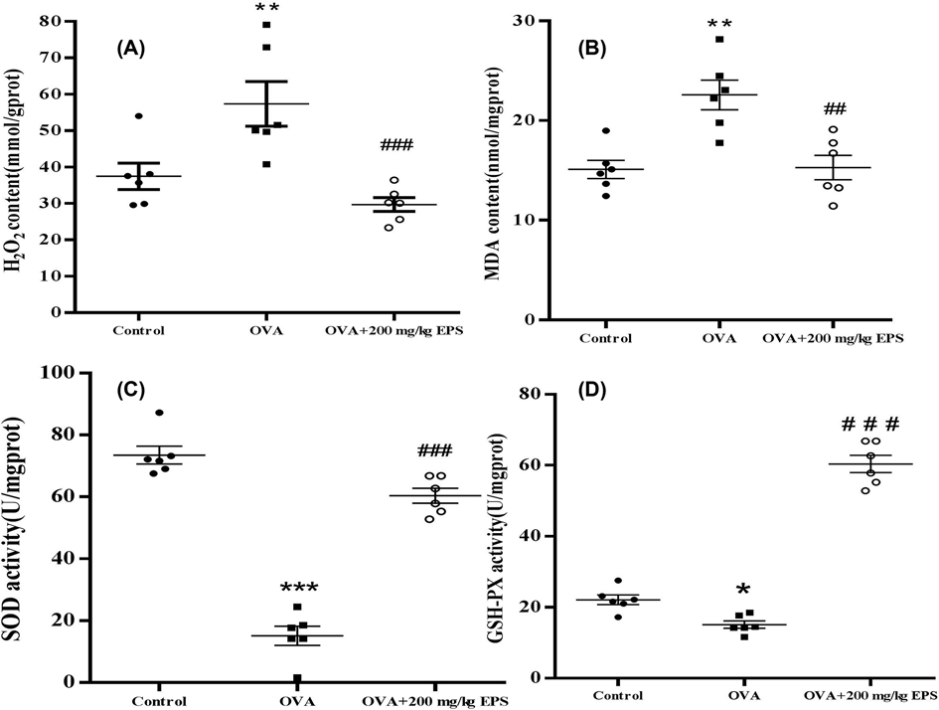
Effects of dexamethasone and the EPS on antioxidant indexes in BALF in the lungs of mice in each group (**A**) H_2_O_2_ content, (**B**) MDA content, (**C**) SOD activity, and (**D**) GSH-PX. Data are expressed as the mean ± standard error (*n*=6). *P<0.5, **P<0.01, ***P<0.001 versus control; ^##^*P*<0.01, ^###^*P*<0.001, versus the OVA group.

It has been reported that the EPS from *B. subtilis* has antioxidant activity and inhibited cancer cell growth *in vitro* [21,22]. To test antioxidant activity of the EPS from *B. subtilis* xztubd1 *in vivo*, we evaluated the alleviation of the OVA-induced lung oxidative damage indexes in the 200 mg/kg/day EPS treatment group. As shown in Figure 4, the H_2_O_2_ and MDA contents were observably increased in the lungs of asthmatic mice, whereas the activities of SOD and GSH-PX were markedly decreased in asthmatic mice. Furthermore, the EPS treatment significantly decreased the H_2_O_2_ and MDA levels, dramatically enhanced the SOD and GSH-PX activities compared with OVA alone. These results suggest that the EPS may alleviate reactive oxygen species (ROS)-induced inflammation by removing freeoxygen radicals and boosting antioxidant enzymes.

### Expression of Th2 cytokines and NF-κB and STAT6 pathways components in lungs

**Figure 5:**
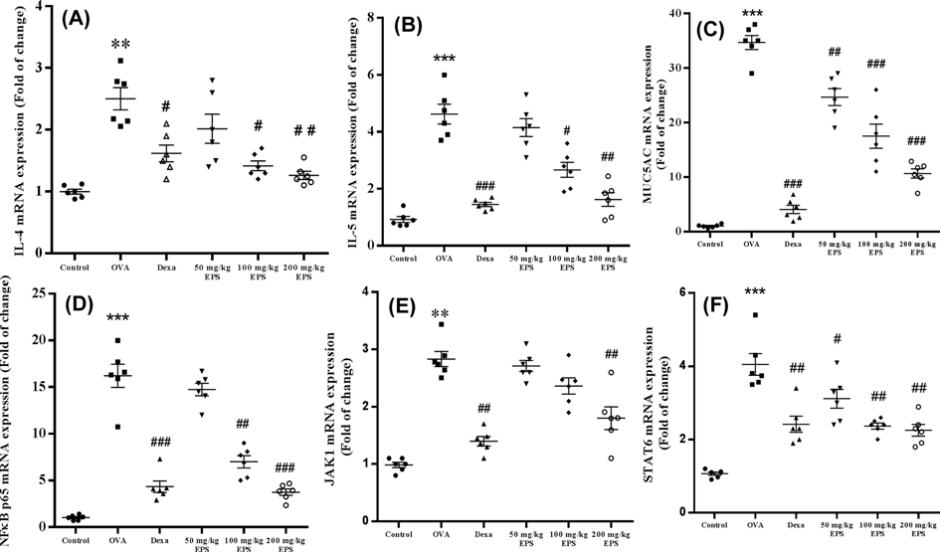
Effects of dexamethasone and the EPS on mRNA levels of Th2 cytokines and components of the NF-κB and STAT6 pathways in the lungs of asthmatic mice (**A**) IL-4, (**B**) IL-5, (**C**) MUC5AC, (**D**) NF- κB p65, (**E**) JAK1, (**F**) STAT6 in the lungs of mice in each group. All values are expressed as fold changes compared with the mean value of the control group. Data are expressed as the mean ± standard error (*n*=6). **P<0.01, ***P<0.001 versus control; ^#^*P*<0.05, ^##^*P*<0.01, ^###^*P*<0.001 versus the OVA group.

**Figure 6:**
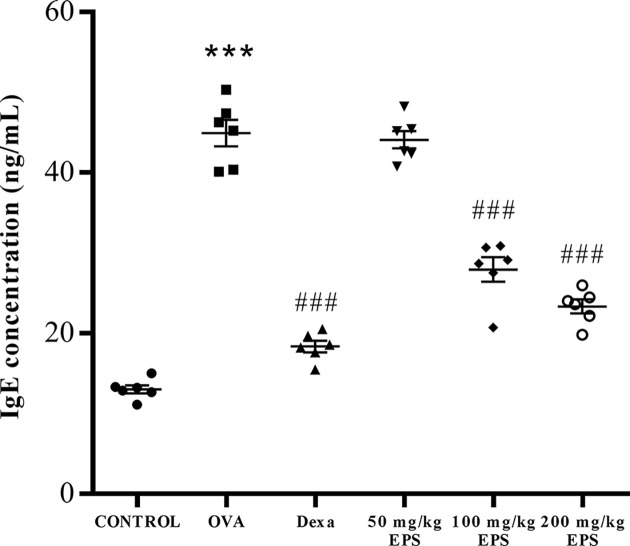
Effects of dexamethasone and the EPS on total serum IgE levels in each group Data are expressed as the mean ± standard error (*n*=6). ***P<0.001 versus control; ^###^*P*<0.001 versus the OVA group.

To further clarify which inflammatory cytokines and signaling pathways might be involved in anti-inflammatory activity of EPS, we examined the levels of Th2 cytokines, MUC5AC, and components of the NF-κB and STAT6 pathways. The OVA group showed markedly elevated IL-4, IL-5, MUC5AC, NF-κB p65, JAK1 and STAT6 expression in the lungs compared with the control mice, whereas their expressions were dramatically reduced in the dexamethasone and OVA+EPS treatment in a dose-dependent manner compared with the OVA group (Figure 5). In Figure 6, total serum IgE is shown to be significantly increased in the OVA treatment relative to the control, while the decrease in the total IgE levels in the serum of mice in the OVA+dexamethasone and OVA+EPS treatment groups (100 and 200 mg/kg) was significant compared with the mice exposed to OVA alone. Overall, these results suggest that the remission of asthmatic inflammatory responses upon EPS treatment is related to the down-regulation of the NF-κB and STAT6 pathways, blocking the Th2 cytokines and subsequent IgE.

## Discussion

In the present study, an OVA-induced asthma model was used to examine how the EPS from *B. subtilis* xztubd1, a novel probiotic, alleviates asthmatic inflammation. Our results showed that the EPS treatment remediated histopathological damage, reduced the number of total inflammatory cells and other cells (e.g., lymphocytes, eosinophils), and increased the macrophage count in the BALF. Th2 cytokines such as IL-4 and IL-5 were diminished and NF-κB and STAT 6 pathway components were down-regulated. Our results revealed a potential mechanism for the EPS attenuation of asthma involving the NF-κB and STAT 6 pathways.

*B. subtilis*, a probiotic, provides many advantages to hosts, such as biofilm formation, the secretion of antimicrobials, the prevention of inflammatory diseases by the modulation of the innate immune response and the T cell-mediated immune response [23,24]. EPSs, important high-molecular weight homo- or heterobiopolymers secreted by microorganisms, have become a very popular topic because of their various biological activities [25,26]. Moreover, the EPS from *B. subtilis* showed antioxidant activity, anticoagulant efficacy, and antitumor activity [27]. Interestingly, the experimental *B. subtilis* xztubd1 was isolated from a housefly active in the winter in the laboratory and identified as *B. subtilis* (GenBank: MG458322.1).

Asthma, a common inflammatory respiratory disease, is characterized by the hypersecretion of mucus and recruitment of inflammatory cells to the airways [20]. We demonstrated obvious alleviation effects from the EPS treatment (200 mg/kg) on asthmatic airway inflammation. The EPS treatment markedly reduced inflammatory cell infiltration, which was demonstrated by a histopathological analysis and the change in the numbers of inflammatory cells in the BALF. Remarkably, the counts of macrophages were significantly increased in EPS treatment, unlike those of lymphocytes, and eosinophils. Accumulating evidence suggests that macrophages are regulators of the lung immune system and the dominant effector cells in innate immune responses [28,29]. It has been reported that EPS from *B. subtilis* can induce M2 macrophage development and specifically bind peritoneal macrophages to protect from intestinal inflammation [11,19]. These findings indicated that the EPS alleviated inflammatory response may occur by the induction of macrophage proliferation and the subsequent production of cytokines.

Th2-mediated inflammatory responses play critical roles in the pathogenesis of asthma by releasing cytokines such as IL-4 and IL-5. IL-4 contributes to the production of the IgE in B lymphocytes and IL-5 plays an important role in allergic airway eosinophilic inflammation [30]. Our results showed that the EPS significantly reduced the lymphocyte count and expression of IL-4, IL-5, and MUC5AC in asthmatic mice. These findings suggest that the EPS alleviated airway eosinophilic inflammation by decreasing Th2-associated cytokines. Additionally, ROS have been associated with airway inflammation in asthma and are known as important regulators of physiological cell signaling [31]. ROS, such as H_2_O_2_, induce the expression of pro-inflammatory cytokines, such as IL-4, involving the activation of the IL-4/STAT6 pathway [32]. Our results showed that EPS treatment decreased the H_2_O_2_ and MDA contents, increased SOD and GSH-PX activities and reduced the expression of IL-4 and IL-5, which further eliminated ROS-induced inflammation. These findings implied that EPS treatment reduced airway inflammation through the inhibition of Th2 cytokine expression, partially via the lowering of ROS and increasing antioxidative enzymes.

The JAK/STAT6 pathway has been reported to regulate pathological characteristics in asthmatic animal models, such as Th2 cell differentiation, and airway eosinophilia [33]. The NF-κB inflammatory pathway could amplify the inflammatory response and aggravate asthma symptoms [34]. Our results showed that the EPS treatment significantly reduced the expression of JAK1, STAT6, and NF-κB in the lungs of asthmatic mice. Our findings suggested that the EPS treatment attenuated inflammation by the NF-κB and STAT6 pathways.

In summary, our results suggest that EPS from *B. subtilis* xztubd1 prevented OVA-induced asthmatic airway inflammation via the NF-κB and STAT6 pathways through inhibition of the release of Th2 cytokines and the reduction in ROS.

## Conclusion

The results of the present study provide evidence that the EPS from *B. subtilis* xztubd1 attenuates Th2 cell-mediated inflammation via the NF-κB and STAT6 pathways in the airway of asthmatic mice. The EPS treatment alleviated pulmonary pathological changes, significantly decreased inflammatory cell numbers, other than an increase in macrophages, reduced the H_2_O_2_ and MDA contents, increased the SOD and GSH-PX activities, down-regulated the expression of components of the NF-κB and STAT6 pathways and Th2 cytokines (IL-4, IL-5). Understanding the mechanism underlying the effects of the EPS is not only important for the development of therapeutic strategies for asthma but also provides a scientific basis for developing new drugs for asthma.

## Data Availability

Data and materials are available upon request.

## Abbreviations

BALF: bronchoalveolar lavage fluid
EPS: exopolysaccharide
GSH-PX: glutathione peroxidase
HE: hematoxylin–eosin
H_2_O_2_: hydrogen peroxide
IL: interleukin
MDA: malondialdehyde
MUC5AC: mucin 5AC
NF-κB: nuclear factor-κB
OVA: ovalbumin
ROS: reactive oxygen species
SOD: superoxide dismutase
Th2: type II T-helper cell
